# Developing sensitive quality indicators for ground inter-hospital transport of adult critically ill patients using Donabedian model

**DOI:** 10.3389/fpubh.2026.1744809

**Published:** 2026-04-14

**Authors:** Qianqian Zhang, Ge Zang, Naifu Tang, Li Zhang, Manman Yin, Tongtong Zhang, Bo Li

**Affiliations:** 1 Department of Emergency Medicine, The First Affiliated Hospital of Zhengzhou University, Zhengzhou, China; 2 Nursing Department, The First Affiliated Hospital of Zhengzhou University, Zhengzhou, China

**Keywords:** critically ill patients, Delphi method, Donabedian model, inter-hospital transport, quality indicators

## Abstract

**Background:**

The complexity of inter-hospital transport (IHT) for critically ill patients poses challenges to quality evaluation. Currently, sensitive quality indicators for the entire process of IHT remain lacking. The objective of this study is to develop a set of standardized and quantifiable quality evaluation indicators for land-based IHT of adult critically ill patients.

**Methods:**

Based on the structure-process-outcome three-dimensional model, we developed the first-round Delphi questionnaire through literature review, semi-structured interviews, and research group discussions. We invited nursing, medical, and administrative experts in emergency medicine, critical care, and IHT from various institutions to participate in two rounds of Delphi consultation, and established the quality evaluation indicator system for IHT. The indicator weights were determined using the Analytic Hierarchy Process (AHP).

**Results:**

The response rates for the two rounds of the expert Delphi questionnaire were 100 and 95.83%, respectively. The proportion of experts providing suggestions was 83.3% in the first round and 21.7% in the second round. The expert authority coefficient (*Cr*) was 0.950 for the first round and 0.974 for the second round. The Kendall’s concordance coefficients (Kendall’s *W*) for the third-level indicators were 0.298 (first round) and 0.327 (second round), both of which were statistically significant (*p* < 0.001). Through this expert consultation process, a finalized quality indicator system was established, comprising 3 first-level indicators, 10 s-level indicators, and 22 third-level indicators.

**Conclusion:**

The sensitive quality indicators for land-based IHT of adult critically ill patients developed in this study enable comprehensive quality monitoring of the entire transfer process through the dimensions of structure, process, and outcome. Those quality indicators provide a basis for objectively evaluating the quality of IHT, identifying weaknesses in transport practice, and improving transport quality.

## 1 Introduction

Inter-hospital transport (IHT) refers to the transfer of a patient from one medical facility to another. Patients are usually transferred to higher-level hospitals with more advanced resources and specialized expertise. This transfer may improve patient clinical outcomes. IHT is a relatively complex clinical practice process. These transfers often involve long distances and are time-consuming (1). It requires collaboration between multiple healthcare facilities. A typical IHT often involves three key entities: the referring facility, the transporting unit, and the receiving facility. In China, the transporting unit may be performed by the referring hospital, the receiving hospital, a professional transport institution, or other entities (2). With the comprehensive implementation of China’s tiered healthcare system, transferring critically ill patients to hospitals with superior treatment capabilities has become common practice.

IHT faces multiple challenges, including inconsistent levels of expertise among healthcare personnel, varying availability of resources, and the absence of standardized communication protocols between referring and receiving facilities (3). When moving from a familiar hospital environment to an ambulance setting with limited support, nurses often encounter numerous challenges during land IHT, such as unclear responsibility demarcation, inadequate adaptation to the ambulance environment, and lack of insight during transport. Survey unclear responsibility demarcation, insufficient adaptation to the ambulance environment, and lack of insight during transport (4). Notably, patients face inherent risks of clinical deterioration during transport. According to the large Ground and Air Medical Quality Transport (GAMUT) database analysis, the incidence of cardiopulmonary resuscitation during IHT was 0.42% (5). Although cardiopulmonary resuscitation events are relatively rare, the rate of return of spontaneous circulation was only 46.9% (6). In neonatal IHT, real-time tracking of transport safety data can reduce safety errors (7). The application of scientific quality management methods can enhance patient safety during IHT, such as the implementation of quality indicators. The implementation of quality indicators can encourage personnel to proactively take protective measures during transport (8).

Nevertheless, current research on the quality assessment of IHT has focused primarily on single outcome measures, such as the incidence of specific adverse events (9). The GAMUT Quality Improvement Collaborative collects 40 IHT quality indicators, primarily covering aspects such as advanced airway management, blood products, ventilator management, efficiency, maternal transport, and safety events (10). However, IHT involves multi- institutional collaboration and complex decision-making processes, these indicators fail to fully reflect the multiple factors influencing the complex process. Therefore, sensitive quality indicators for evaluating the entire process of inter-hospital transport are still lacking, and such indicators need to be developed under the guidance of a standardized framework.

Donabedian three-dimensional quality structure model has been applied to the development of quality assessment indicator systems (11), such as the formulation of nursing indicators for patients receiving prone position ventilation (12). This study aims to develop a quality assessment tool for adult ground-based IHT, framed by Donabedian structure-process-outcome model, so as to evaluate the entire process and key components of IHT.

## 2 Methods

### 2.1 Design

**Figure 1:**
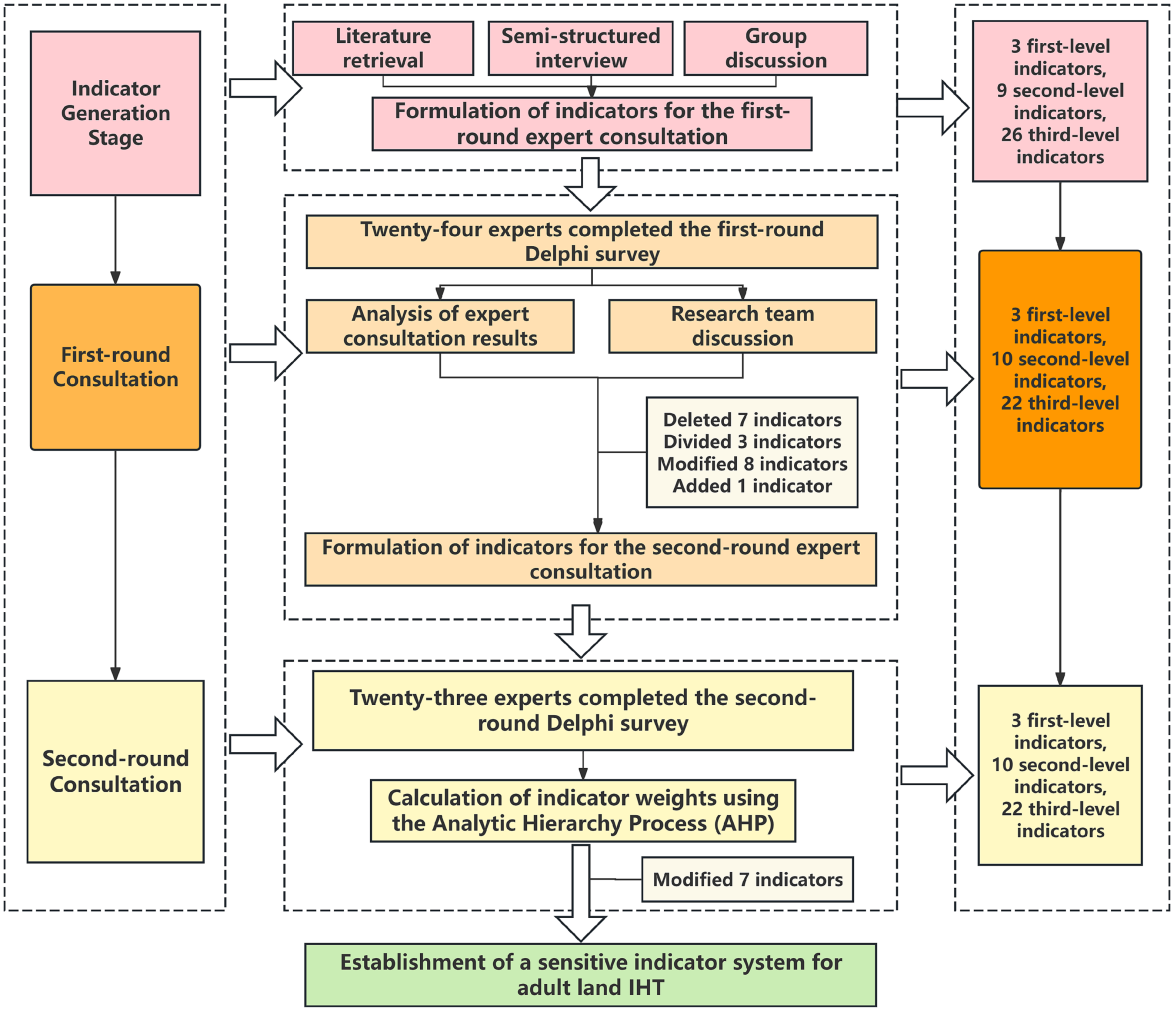
Flowchart of quality indicator development for IHT.

Based on Donabedian structure-process-outcome theoretical model, the quality index of IHT in critically ill adult patients was initially established through literature search and semi-structured interview. The Delphi survey was conducted from June to September 2024, among 24 nursing, medical and management specialists in the fields of emergency, intensive care and IHT. The process for developing IHT quality indicators is shown in Figure 1.

### 2.2 The establishment of a research team

The research team consisted of 14 members. There were six doctors and eight nurses. The team comprised 3 head nurses and 3 directors from the departments of emergency, intensive care or IHT. In the team, there were 2 IHT doctors, 1 intensive care doctor, 2 IHT nurses, 2 emergency nurses, and 1 intensive care nurse engaged in the clinical front line. In terms of academic qualifications, the research team consisted of 1 member with a doctoral degree, 8 with master’s degrees, and the remainder with bachelor’s degrees. The main responsibilities of the research team were to search and sort literature, formulate a semi-structured interview outline and conduct interviews, preliminarily draw up quality evaluation indicators for adult IHT, compile correspondence questionnaires, select correspondence experts, and issue, collect and sort out correspondence questionnaires.

### 2.3 Data collection

#### 2.3.1 Literature source and retrieval method

We used “inter-hospital,” “interfacility,” “transport,” “transfer,” “referral,” “critically ill patient,” “critical care,” “critical illness,” “quality indicators,” “quality indexes,” “quality evaluation,” “quality safety,” and “evaluation indicators” as keywords. Literature searches were conducted in PubMed, Embase, Scopus, Cochrane Library, and Chinese databases including the CNKI database, Wanfang data, Wanfang Medical online. Chinese and English literature from the establishment of the database to March 2024 was retrieved. Using 12 articles as the reference basis (2, 5, 13–22), we retrieved the quality indicators from the GAMUT Quality Improvement Collaborative (10), and developed 29 preliminary indicator items (Appendix 1 in Supplementary material).

#### 2.3.2 Semi-structured interviews

A semi-structured interview was conducted with 8 emergency department directors, head nurses, nursing team leaders, and medical team leaders from hospitals providing IHT services. Three inclusion criteria were established for interviewees. Participants were required to meet either criterion (1) or (2), in addition to criterion (3): (1) The emergency department director or head nurse had been employed for 3 years or more; (2) The nursing team leader and medical team leader had at least 5 years of emergency work experience and participate in IHT at least 10 times per year; (3) All participants had relevant experience in quality control management. All participants were asked questions regarding IHT quality management, including the most important aspects of IHT, the most difficult challenges encountered during IHT implementation, and the key sensitive indicators for evaluating IHT quality. Finally, the researchers sorted out and analyzed the interview data. Nine relevant themes were extracted, including organization and management, human resource management, pre-transport assessment, transport communication, the practice of transport process, safety management, and so on(Appendix 2 in Supplementary material).

#### 2.3.3 Group discussion

The research team discussed the indicators and themes obtained from the literature search and semi-structured interviews, removed three indicators that overlapped with others, and then classified the indicators according to the Donabedian model. Following group discussions, consensus was reached on indicator nomenclature, calculation methodologies, and data collection procedures, leading to the development of the first-round Delphi questionnaire. This questionnaire comprised 3 first-level indicators, 9 s-level indicators, and 26 third-level indicators.

#### 2.3.4 Delphi method

##### 2.3.4.1 Design the Delphi survey correspondence questionnaire

The first-round questionnaire included three parts, which were preface, indicator correspondence and basic information of experts. The preface introduced the significance of the research, the components of the questionnaire, the time of sending back the questionnaire and the points for attention. The indicator correspondence form mainly comprised indicators, the indicator definition and connotation of indicators, the calculation formula of indicators, the collection method of data, and the importance score of indicators. In addition, the correspondence form had a suggestion column for improving indicators and a suggestion column for adding indicators. Experts could also choose to retain, modify, or delete the indicator in the correspondence form. The experts’ basic information contained the personal information, the basis of their judgments, and their degree of familiarity with the research topic.

##### 2.3.4.2 Delphi expert correspondence consultation

Twenty-four experts from professional and authoritative Grade III Level A hospitals and 120 centers in 11 provinces and cities of China were selected for correspondence consultation. These experts were from the professional fields of pre-hospital emergency, emergency, critical care, and critical care transport. Regarding the selection criteria for specialists, the head nurse and department director have more than 5 years of relevant management experience. Doctors and nurses must have at least 10 years of relevant clinical experience. In addition, the professional title of all experts should be intermediate or above. Furthermore, all participating experts should have at least a bachelor’s degree. Two rounds of expert consultations were conducted from June to September 2024. Before issuing the questionnaire, the researcher contacted the experts via telephone or WeChat to obtain informed consent. Subsequently, the questionnaires were disseminated to participating experts through email and printed formats. The research team sorted out and analyzed the questionnaires, and retained the items with an average importance score > 3.5 and a coefficient of variation (CV) < 0.25. Meanwhile, indicators were modified, deleted and supplemented according to the expert written opinions.

#### 2.3.5 Preliminary test indicators

A convenience sampling method was adopted to enroll critically ill patients who underwent IHT by a tertiary Grade A hospital in Henan Province, China, in September 2025. This hospital has a full-time dedicated IHT team. Based on some of the third-level indicators in the developed quality indicator system, “The Questionnaire on Quality Indicators for Adult Inter-Hospital Transport” was designed. The questionnaire consists of three sections completed, respectively, by nurses, patients or their families, and the quality control group. The sections completed by nurses and patients or their families were filled out during the transport process, whereas the section for the quality control group was completed weekly. Before data collection, the research team provided training for transport staff and quality control group on indicator interpretation and standardized data reporting methods.

### 2.4 Statistical analysis

Data entry and statistical analyses were performed using Excel 2019, SPSS 23.0, and SPSS AU software. Expert enthusiasm was measured by the questionnaire response rate, with a response rate exceeding 70% indicating a high level of expert enthusiasm. Expert authority was represented by the authority coefficient (Cr), which was the arithmetic mean of the expert’s familiarity coefficient (Cs) with the consultation content and the judgment basis coefficient (Ca). The calculation formula was Cr = (Cs + Ca)/2. Expert coordination was reflected by Kendall’s W coefficient and the coefficient of variation (CV). Indicator weights were determined through the Analytic Hierarchy Process (AHP). The combined weight values of the third-level indicators were derived from the product of the first-level, second-level and third-level indicator weights. Statistical significance threshold was set at *p* < 0.05.

### 2.5 Ethical considerations

This study adhered to the principles of the Declaration of Helsinki and was formally approved by the Ethics Committee of the First Affiliated Hospital of Zhengzhou University (Ethics Approval No.: 2024-KY-0190).

## 3 Results

### 3.1 Basic information on experts

The 24 experts consulted in this study were from 16 hospitals and 2 emergency centers in 11 provinces and cities in China. The age of the experts consulted in the first round ranged from 35 to 59 years old, with an average age of (44.00 ± 5.26) years. The experts came from the professional fields related to undertaking and participating in the task of IHT of critical patients, including 7 in the field of critical care transport (29.17%). The working life of experts in these fields ranges from 10 years to 30 years, with an average of (19.00 ± 6.09) years. Among the experts interviewed by letter, 18 were head nurses and directors (75.0%), and 6 were doctors and nurses (25.0%). There were 20 senior and associate senior professional titles (83.3%) and 4 intermediate professional titles (16.7%). The general demographic data of experts in two rounds of consultation are shown in Table 1.

**Table 1:** Demographic data of experts consulted.

Variables	Round 1 (*n* = 24)	Round 2 (*n* = 23)
	*n* (%)	*n* (%)
Gender		
Male	10 (41.67)	9 (39.13)
Female	14 (58.33)	14 (60.87)
Professional title		
Senior professional title	8 (33.33)	7 (30.44)
Associate senior professional title	12 (50.00)	12 (52.17)
Intermediate professional title	4 (16.67)	4 (17.39)
Educational background		
Doctorate degree	3 (12.5)	3 (13.04)
Master’s degree	10 (41.67)	9 (39.13)
Bachelor’s degree	11 (45.83)	11 (47.83)
Professional field		
Critical care transport field	7 (29.17)	7 (30.44)
Pre-hospital emergency field	4 (16.67)	3 (13.04)
Emergency department field	6 (25.00)	6 (26.09)
Critical care field	7 (29.17)	7 (30.44)
Relevant professional work experience		
10–20 years	16 (66.67)	16 (69.57)
>20 years	8 (33.33)	7 (30.44)
Provinces		
Henan	8 (33.33)	7 (30.44)
Beijing	3 (12.50)	3 (13.04)
Zhejiang	3 (12.50)	3 (13.04)
Chongqing	2 (8.33)	2 (8.70)
Jiangsu	2 (8.33)	2 (8.70)
Fujian	1 (4.17)	1 (4.35)
Gansu	1 (4.17)	1 (4.35)
Guangdong	1 (4.17)	1 (4.35)
Hubei	1 (4.17)	1 (4.35)
Hunan	1 (4.17)	1 (4.35)
Sichuan	1 (4.17)	1 (4.35)

### 3.2 The degree of initiative, authority and coordination of experts

In this study, 24 consultation questionnaires were distributed in each of the two rounds of expert consultation. Twenty-four questionnaires were effectively collected in the first round, and 23 questionnaires were effectively collected in the second round, with effective recoveries of 100 and 95.83%, respectively. Twenty experts made recommendations in the first round of expert consultations (83.3%), 5 experts made recommendations in the second round (21.7%), indicating that the experts were highly motivated to participate in the content of this study. The judgment basis, familiarity degree, and authority coefficient of the two rounds expert consultation are shown in Table 2. In addition, the Kendall harmony coefficients of the third-level indicators in the two rounds of expert consultation were 0.298 and 0.327, respectively, and the differences were statistically significant (Table 3).

**Table 2:** The judgment basis, familiarity degree, and authority coefficient of the two rounds expert consultation.

Round	Ca^a^	Cs^b^	C^c^
First-round	0.979	0.921	0.950
Second-round	0.991	0.957	0.974

a Ca: judgment basis.

b Cs: familiarity coefficient.

c Cr: authority coefficient. A higher Cr value indicated a higher level of expert authority. A Cr value > 0.7 indicated a high level of expert authority.

**Table 3:** Degree of coordination between the two rounds expert consultation.

Items	Indicators	Kendall *W*^a^	*X*^2^ ^b^	*p*
First round				
Second-level indicators	9	0.114	21.992	0.005
Third-level indicators	26	0.298	178.635	0.000
Second round				
Second-level indicators	10	0.126	26.118	0.002
Third-level indicators	22	0.327	157.88	0.000

a *W*: The value of Kendall’s *W* ranged from 0 to 1, with a higher value indicating a higher level of consensus among experts.

b *P*: A chi-square test showed that a *p*-value< 0.05 indicated significant agreement among experts in their ratings.

### 3.3 Results of expert consultations

#### 3.3.1 The first-round of expert consultation

In the first-round consultation, experts evaluated the preliminary draft of quality indicators for land-based IHT of adult critically ill patients (Table 4). According to the indicator screening criteria, combined with experts’ opinions and the research team’s discussion results, in the first-round expert consultation, 7 indicators were deleted, 3 indicators were divided, 8 indicators were modified, and 1 indicator was added. Because the CV values of “transport cancellation rate after transport team start-up” and “mortality within 24 h of admission” were greater than 0.25, they were finally deleted after discussion by the research team based on expert opinions. The adjustments to quality indicators after the first-round of the Delphi survey are detailed in Table 5.

**Table 4:** Results of the first-round of the Delphi survey.

First-level indicators	Second-level indicators	Third-level indicators	Mean (*SD*)	*CV*^c^	Proportion of full scores (%)
I structure	I-1 Organization and management^a^		4.71 ± 0.69	0.147	83.3%
		I-1.1 Vehicle equipment and drug integrity rate ^a^	4.96 ± 0.20	0.041	95.8%
		I-1.2 Established inter-hospital transport process and emergency plan^a^	4.96 ± 0.20	0.041	95.8%
	I-2 Human resource management		4.88 ± 0.45	0.092	91.7%
		I-2.1 Qualification rate of the transport team assessment	4.83 ± 0.48	0.100	87.5%
		I-2.2 Qualification rate of transport team configuration	4.79 ± 0.51	0.106	83.3%
II process	II-1 Pre-transport assessment		4.96 ± 0.20	0.041	95.8%
		II-1.1 Complete rate of initial assessment before driving ^b^	4.83 ± 0.48	0.1	87.5%
		II-1.2 Planning rate of the best route for transport ^b^	4.33 ± 0.92	0.212	58.3%
		II-1.3 Accuracy rate of disease assessment classification	4.87 ± 0.34	0.069	87.5%
		II-1.4 Accuracy rate of transport risk assessment classification	4.71 ± 0.86	0.182	83.3%
	II-2 Transport communication		4.79 ± 0.42	0.087	79.2%
		II-2.1 Completeness rate of signing the inter-hospital transport informed consent	4.87 ± 0.34	0.069	87.5%
		II-2.2 Efficient communication between transfer out—transport—receiving units ^b^	4.79 ± 0.42	0.087	79.2%
	II-3 The practice of transport process		4.79 ± 0.42	0.087	79.2%
		II-3.1 Implementation rate of dynamic disease evaluation	4.79 ± 0.51	0.106	83.3%
		II-3.2 Correct handling rate of changes in condition during transport ^a^	4.83 ± 0.82	0.169	95.8%
		II-3.3 Accurate execution rate of medical orders	4.67 ± 0.92	0.196	83.3%
		II-3.4 Qualified rate of analgesic management ^b^	4.08 ± 0.88	0.216	37.5%
		II-3.5 Qualified rate of sedation management ^b^	4.08 ± 0.83	0.203	33.3%
		II-3.6 Completion rate of transport handover ^a^	4.92 ± 0.28	0.057	91.7%
		II-3.7 Qualified rate of medical documentation for transport	4.67 ± 0.57	0.121	70.8%
III result	III-1 Safety management ^a^		5.00 ± 0.00	0	100%
		III-1.1 Incidence of disease exacerbations ^a^	4.67 ± 0.57	0.121	70.8%
		III-1.2 Incidence of mobility problems ^a^	4.58 ± 0.65	0.143	66.7%
		III-1.3 Incidence of technical problems ^a^	4.67 ± 0.57	0.121	70.8%
	III-2 Service quality		4.71 ± 0.55	0.117	75%
		III-2.1 Patient satisfaction with transport medical services	4.42 ± 0.78	0.176	58.3%
		III-2.2 Transport cancellation rate after transport team start-up ^b^	3.42 ± 1.32	0.385	25%
	III-3 Transport timeliness		4.71 ± 0.55	0.117	75%
		III-3.1 Average time taken to prepare for transport	4.54 ± 0.59	0.130	58.3%
		III-3.2 Median transport stabilization time	4.29 ± 0.86	0.200	50%
	III-4 Patient outcome^a^		4.71 ± 0.55	0.117	75%
		III-4.1 Incidence of increase in disease assessment grade of patients after transport ^a^	4.33 ± 1.01	0.233	54.2%
		III-4.2 Mortality within 24 h of admission ^b^	3.87 ± 1.15	0.298	37.5%

a Indicates the indicator requires division or revision.

b Indicates the indicator requires deletion.

c CV: A smaller CV value indicated less divergence of expert opinions on the corresponding indicator. A CV <0.25 indicated a relatively high level of expert consensus on the indicator.

**Table 5:** Quality indicator adjustments after the first-round of the Delphi survey.

Indicator level	Original indicators	Form of adjustment	Revised indicators
Second-level	I-1 Organization and management	Division	I-1 System and process
			I-2 Material management
Second-level	III-1 Safety management	Revision	III-1Adverse event
Second-level	III-4 Patient outcome	Revision	III-4 Transport effectiveness
Third-level	I-1.1 Vehicle equipment and drug integrity rate	Division	I-2.1 Vehicle equipment integrity rate
			I-2.2 Vehicle drug integrity rate
Third-level	I-1.2 Established inter-hospital transport process and emergency plan	Division	I-1.1 Develop inter-hospital transfer procedures
			I-1.2 Formulate emergency plans for inter-hospital transport emergencies and conduct regular drills
Third-level	II-1.1 Complete rate of initial assessment before driving ^b^	Deletion	
Third-level	II-1.2 Planning rate of the best route for transport ^b^	Deletion	
Third-level	II-2.2 Efficient communication between transfer out—transport—receiving units ^b^	Deletion	
Third-level	II-3.2 Correct handling rate of changes in condition during transport	Revision	II-3.2 Effective treatment rate of changes in condition during transport
Third-level	II-3.4 Qualified rate of analgesic management ^b^	Deletion	
Third-level	II-3.5 Qualified rate of sedation management ^b^	Deletion	
Third-level	II-3.6 Completion rate of transport handover	Revision	II-3.4 The implementation rate of standardized handover
Third-level	III-1.1 Incidence of disease exacerbations	Revision	III-1.1 Tube blockage and accidental displacement rate of treatment device
Third-level	III-1.2 Incidence of mobility problems	Revision	III-1.2 Incidence of medical equipment failure during transport
Third-level	III-1.3 Incidence of technical problems	Revision	III-1.3 Incidence of patient injury related to transport
Third-level	III-2.2 Transport cancellation rate after transport team start-up ^a^	Deletion	
Third-level	III-4.1 Incidence of increase in disease assessment grade of patients after transport	Revision	III-4.1 Beneficial rate of patient clinical outcome after transport
Third-level	III-4.2 Mortality within 24 h of admission ^a^	Deletion	
Third-level	Rate of compliance with the expected arrival time of transport	Addition	

a Indicators were deleted due to CV > 0.25.

b Indicators were deleted based on expert opinions.

#### 3.3.2 The second-round of expert consultation

After the second round of expert consultation, one second-level indicator and six third-level indicators were modified. The second-level indicator “human resource management” was modified to “personnel management.” “Formulate emergency plans for IHT emergencies and conduct regular drills” is amended to “formulate emergency plans for IHT emergencies.” “Qualified rate of transport team staffing” was modified to “qualified rate of hierarchical configuration of transport team members.” “Completeness rate of signing the IHT informed consent” was revised to “standard signing rate of informed consent for IHT.” “Tube blockage and accidental displacement rate of treatment device” was changed to “Incidence of accidental catheter displacement.” “Incidence of patient injury related to transport” was revised to “incidence of patient accidental injuries during transport.” “Beneficial rate of patient clinical outcome after transport” was modified to “rate of transport patients smoothly arrive.” After the second-round of expert consultation, the quality indicator system was formed, including 3 first-level indicators, 10 s-level indicators and 22 third-level indicators. The importance scores of the indicators were in the range (4.30 ~ 5.0), and the CV values were in the range of (0 ~ 0.15). The AHP method was used to calculate the indicator weights, and the quality indicator system and indicator weights are shown in Table 6. Definitions and explanations of the third-level indicators are presented in Appendix 3 in Supplementary material.

**Table 6:** Results of the second-round of the Delphi survey.

First-level indicators	Second-level indicators	Third-level indicators	Mean (*SD*)^a^	Combined weight^b^
I Structure (0.285)	I-1 System and process (0.325)	I-1.1 Develop inter-hospital transport procedures (0.558)	5.0 ± 0.0	0.052
		I-1.2 Formulate emergency plans for inter-hospital transport emergencies (0.443)	4.96 ± 0.209	0.041
	I-2 Material management (0.329)	I-2.1 Vehicle equipment integrity rate (0.590)	4.96 ± 0.209	0.055
		I-2.2 Vehicle drug integrity rate (0.410)	5.0 ± 0.0	0.038
	I-3 Personnel management (0.346)	I-3.1 Qualified rate of examination of transport team members (0.550)	5.0 ± 0.0	0.054
		I-3.2 Qualified rate of hierarchical configuration of transport team members (0.451)	5.0 ± 0.0	0.045
II Process (0.368)	II-1 Pre-transport assessment (0.295)	II-1.1 Accuracy rate of disease assessment classification (0.539)	5.0 ± 0.0	0.059
		II-1.2 Accuracy rate of transport risk assessment classification (0.461)	5.0 ± 0.0	0.050
	II-2 Transport communication (0.119)	II-2.1 Standard signing rate of informed consent for inter-hospital transport (0.044)	4.78 ± 0.518	0.044
	II-3 The practice of transport process (0.586)	II-3.1 Implementation rate of dynamic disease evaluation (0.227)	5.0 ± 0.0	0.049
		II-3.2 Effective treatment rate of changes in condition during transport (0.240)	4.96 ± 0.209	0.052
		II-3.3 Accurate execution rate of medical orders (0.189)	4.78 ± 0.518	0.041
		II-3.4 The implementation rate of standardized handover (0.192)	5.0 ± 0.0	0.041
		II-3.5 Qualified rate of medical documentation for transport (0.152)	5.0 ± 0.0	0.033
III result (0.346)	III-1 Adverse events (0.381)	III-1.1 Incidence of accidental catheter displacement (0.344)	4.91 ± 0.288	0.046
		III-1.2 Incidence of medical equipment failure during transport (0.270)	4.96 ± 0.209	0.036
		III-1.3 Incidence of patient accidental injuries during transport (0.386)	4.91 ± 0.288	0.051
	III-2 Service quality (0.118)	III-2.1 Patient satisfaction with transport medical services (0.041)	4.3 ± 0.559	0.041
	III-3 Transport timeliness (0.268)	III-3.1 Average time taken to prepare for transport (0.354)	5.0 ± 0.0	0.033
		III-3.2 Median transport stabilization time (0.337)	4.61 ± 0.499	0.031
		III-3.3 Rate of compliance with the expected arrival time of transport (0.310)	4.57 ± 0.662	0.029
	III-4 Transport effectiveness (0.232)	III-4.1 Rate of transport patients smoothly arrive (0.080)	4.61 ± 0.583	0.080

a SD, standard deviation.

b The combined weight of a third-level indicator represents its final weight in the overall evaluation of the indicator system, and all combined weights were normalized to sum to 1.

### 3.4 Preliminary test results

A total of 128 quality indicator questionnaires for IHT patients were collected. Five questionnaires were excluded due to incomplete filling by nurses, patients or their families. The effective response rate of the questionnaires was 96.1%. The preliminary test data of quality indicators for IHT are shown in Table 7.

**Table 7:** Preliminary testing of quality sensitive indicators for IHT.

Indicators	Indicator value	Indicators	Indicator value
Incidence of accidental catheter displacement	41‰	The implementation rate of standardized handover	89.4%
Incidence of medical equipment failure during transport	130.1‰	Patient satisfaction with transport medical services	7.95 ± 1.06
Incidence of patient accidental injuries during transport	8‰	Median transport stabilization time	69 (56, 85)
Qualified rate of medical documentation for transport	85.4%	Rate of compliance with the expected arrival time of transport	17.1%
Standard signing rate of informed consent for inter-hospital transport	91.1%	Rate of transport patients smoothly arrive	93.5%

## 4 Discussion

### 4.1 The sensitive quality indicator of land-based IHT in adult critically ill patients is scientific and reliable

Literature has confirmed that transport quality is the weakest link in the critical care patient transport process (23). This study aims to establish a sensitive quality assessment system for land-based IHT of adult critically ill patients. Experts from East China, Southwest China, Northwest China, South China and North China were invited, and the rate of expert opinion was 83.3 and 21.7%, respectively, in the two rounds of expert consultation, indicating that the experts consulted were representative and active. The Cr value of the quantitative evaluation index of expert authority degree was higher than 0.8 in both rounds of consultation, indicating that experts have high authority in the field of acute and critical care IHT. Furthermore, the Kendall harmony coefficient of the two rounds of consultation was 0.298 and 0.327 respectively, and the CV values of each index of the second round of expert consultation were all less than 0.25, indicating that the experts had a good degree of coordination and their opinions were consistent. The Cr value of the consistency test of each level of index weight was less than 0.1, indicating that the weight was reasonable and the results were scientific and reliable.

The quality indicators developed in this study differ from those used by the Non-Emergency Patient Transport Services (NEPTS) of the National Health Service (NHS) and the indicators from The GAMUT Quality Improvement Collaborative. NEPTS collects data across various non-emergency transport categories, yet there are relatively few quality indicators specific to IHT (24). The quality indicators for IHT collected by The GAMUT Quality Improvement Collaborative mainly focus on patients receiving different treatment modalities (10). The present study was limited to the IHT setting, and quality indicators covering the structure, process, and outcome dimensions of IHT were established based on the Donabedian model. These indicators cover a comprehensive assessment of transport, including transport process, transport equipment management, personnel management, the entire transport procedure, transport timeliness, and other related aspects.

### 4.2 Structural indicators mainly include system and process, material management, personnel management

The three secondary indicators of the structural indicators are similarly weighted, with weights ranging from 0.325 to 0.346. It shows that the development of a perfect transport system and process, standardized material and personnel management are the primary conditions to ensure the quality of IHT for patients with acute and critical illnesses. This concurs with Xiuwen Chen’s findings (12). The combination weight of the three three-level indicators of “develop inter-hospital transport procedures,” “vehicle equipment integrity rate,” “qualified rate of examination of transport team members” is the highest. The combined weights of these three indicators are 0.052, 0.055, and 0.054, respectively. A Chinese expert consensus has recommended that IHT should be carried out according to a standardized IHT procedure for patients with acute and critical illnesses (2). However, a survey shows that most of the countries surveyed (63.13%) have no relevant regulations or laws (25). This prompts the manager to establish a standardized transport system and process to ensure that the transport process has rules to follow. Previous literature has also confirmed that well-resourced teams are beneficial for mitigating disease deterioration (26), and rational staffing is the key to adverse event management during transport (27). Nurses with varying levels of experience possess distinct perceptions regarding the safety and risks associated with inter-hospital transfers (28). Patient-related safety incidents are not solely linked to the transport team; in fact, more than half are associated with medical equipment (29). But only 67.7 percent of countries have the medical equipment needed to transport critically ill patients, and nearly half have no specific training for transport personnel (25). It is suggested that sufficient transport equipment should be equipped, schedule maintenance and management of equipment should be strengthened, and professional training and assessment related to IHT should be paid attention to, so that transport personnel can master the theory and skill level required for transport, so that IHT can be carried out safely.

### 4.3 Process indicators mainly include pre-transport assessment, transport communication, the practice of transport process

Process indicators have the most weight among the first-level indicators, and its weight is 0.36835. Process indicators are the core of quality control of IHT. Process quality assessment aims to ensure that medical practices are conducted in the most appropriate manner. Process indicators consist of 3 s-level indicators and 8 third-level indicators. Multi-dimensional quality control is carried out in the process of transport from the aspects of disease severity assessment before transport, risk assessment before transport, signing informed consent, dynamic disease evaluation, standardized handover, and qualified medical documentation. Accurate pre-transport assessment is a prerequisite for ensuring patient safety during transport and can help avoid unnecessary interventions (30). The combination weight of the three-level indicators of “Accuracy rate of disease assessment classification” is the highest, and its weight value” is 0.059. “Accuracy rate of transport risk assessment classification” also has a relatively high combined weight of 0.050. The practice of the transportation process mainly relies on the early clinical assessment within the first few hours before transportation (31), which helps in selecting the appropriate transportation method and equipping the corresponding transportation personnel and equipment. “Effective treatment rate of changes in condition during transport” demonstrates a relatively high weight in this study (0.052), a finding that underscores the critical role of dynamic therapeutic interventions in the comprehensive quality control of IHT processes. Critically ill patients are at risk of clinical deterioration during transport, so professional transport teams are required to implement meticulous management (32). Effective response to changes in the patient’s condition serves as a direct indicator for evaluating the clinical competence of the transport team. In addition, communication with patients and their families becomes a common challenge in IHT, and clear and effective communication is central to timely transports (33). This study used the indicator of “standard signing rate of informed consent for inter-hospital transport” to reflect the communication status in the process of transport.

### 4.4 Result indicators focus on adverse events, service quality, transport timeliness, and transport effectiveness

Result indicators represent the core focus in IHT quality management. As direct feedback on the effectiveness of structural and process implementation, they serve as a critical dimension for evaluating transport efficiency and patient safety. The result indicators developed in this study consist of four second-level indicators and eight third-level indicators. Among them, adverse events represent the second-level indicator with the highest weight among outcome indicators. The GAMUT Quality Improvement Collaborative also regarded adverse events as an important quality indicator for IHT (10), thereby underscoring the core principle of “safety first” in transport practice. The establishment of standardized transport procedures, equipped with professional equipment and qualified transport teams, aims to reduce risks during transport and ensure the safe arrival of patients at the receiving medical facility (34, 35).

Among the third-level indicators, the incidence of patient accidental injuries during transport, the average time taken to prepare for transport, the incidence of accidental catheter displacement, and patient satisfaction with transport medical services were assigned higher weights and combined weights. The incidence of patient accidental injuries during transport (encompassing injuries from traffic accidents, driving errors, falls from stretchers, etc.) and the incidence of accidental catheter displacement (e.g., endotracheal tubes, central venous catheters, drainage tubes) directly quantify the risk of physical harm to patients inherent in the transport process. Such events can readily precipitate severe complications, including secondary injuries, medication extravasation, airway obstruction, and potentially life-threatening situations (36). The average time taken to prepare for transport was assigned a relatively high weight in our indicator system. Prolonged preparation times can delay critical treatment, thereby potentially compromising clinical outcomes. This is particularly critical for time-sensitive conditions such as acute ischemic stroke and acute myocardial infarction (37). Furthermore, both the median transport stabilization time and the rate of compliance with the expected arrival time of transport likewise represent key considerations for ensuring transport safety within comprehensive transport quality management (38). Patients and their families often experience heightened anxiety during transport decision-making and execution. While traditional transport assessments have predominantly focused on technical safety and efficiency, the inclusion of patient satisfaction indicators addresses this gap by providing crucial insight into the humanistic dimensions of care (39). Consequently, patient satisfaction is an indispensable dimension for assessing healthcare service quality. Its high weighting demonstrates that, while safeguarding life remains paramount, enhancing the transport experience and implementing humanistic care are equally critical objectives of high-quality patient transport.

## 5 Limitations

This study has several limitations. The consulted experts were from 11 provinces in China, with a relatively high proportion from central regions. In addition, most of the included experts were from tertiary hospitals, which may result in insufficient representativeness of the expert panel. Further validation is required to determine whether the sensitive indicators developed are applicable in other regions or primary medical institutions. In addition, this study only conducted two rounds of Delphi expert consultation without a third round, which represents a methodological limitation.

## 6 Conclusion

Based on the structure-process-outcome three-dimensional quality model and guided by IHT quality, we developed quality evaluation indicators for adult land IHT through literature review, semi-structured interviews, Delphi expert consultation, and the analytic hierarchy process. This quality indicator system comprises 3 first-level indicators, 10 s-level indicators, and 22 third-level indicators, forming a comprehensive framework. It covers a comprehensive assessment of the entire IHT process. It provides a basis for objectively evaluating the quality of IHT, identifying weaknesses in transport practice, and improving transport quality. The quality indicators were only preliminarily validated in a single hospital with a small sample size. Large-sample and multi-center validation is required in future research to further confirm their reliability and their operability. To further optimize this evaluation tool, we recommend examining the applicability of the indicator system in different regions and hospitals of various levels. Meanwhile, the indicators will be continuously revised and updated based on feedback from healthcare staff during implementation.

## Data Availability

The raw data supporting the conclusions of this article will be made available by the authors, without undue reservation.
