# Supplementary material for: Developing sensitive quality indicators for ground inter-hospital transport of adult critically ill patients using Donabedian model
Source: Front Public Health. 2026 Apr 14;14:1744809. doi: 10.3389/fpubh.2026.1744809 (PMC13121257; doi:10.3389/fpubh.2026.1744809)
Supplement: Supplementary file 1 [file Table_1.doc]

**Appendix 1 The results of literature review**

First, researchers conducted a preliminary screening of articles based on titles and abstracts, followed by a full-text review. Finally, a total of 12 publications were included, comprising 11 English-language articles and 1 Chinese-language article. In addition, indicators from the GAMUT Quality Improvement Collaborative were reviewed. A total of 29 preliminary indicators were extracted from the literature.

**Table S1 Characteristics of Included Literature and Contents of Included Indicators**

| Reference | Literature-based indicator extraction criteria | Extracted indicators |
| --- | --- | --- |
| Bhalala, U. S.; Srivastava, N.; Gothard, M. D.; Bigham, M. T. Cardiopulmonary Resuscitation in Interfacility Transport: An International Report Using the Ground Air Medical Quality in Transport (GAMUT) Database. Crit. Care Res. Pract. 2020, 2020, 4647958. | mobilization time | Ⅲ-3.1 Average time taken to prepare for transport |
| Garrido Conde, B.; Millán García Del Real, N.; Escaplés Giménez, T.; Marsinyach Ros, I.; Toledo Parreño, J. D.; Nuñez Cárdenas, M. D. M.; Domínguez Sampedro, P.; Brandstrup Azuero, K. B. Quality Indicators in Interhospital Transport: Multicentre Project. *An. Pediatr.* 2021, *95* (3), 167–173. | 1. Standardised hand-off  2. Time to stabilization  3. Medication errors  4. Average mobilization time of the transport team  5. Medical equipment failure  6. Patient-centeredness  7. Delays due to resource unavailability | Ⅱ-3.6 Completion rate of transport handover  Ⅲ-3.2 Median transport stabilization time  Ⅱ-3.3 Accurate execution rate of medical orders  Ⅲ-3.1 Average time taken to prepare for transport  Ⅰ-1.1 Vehicle equipment and drug integrity rate  Ⅲ-2.1 Patient satisfaction with transport medical services  Ⅲ-2.2 Transport cancellation rate after transport team start-up |
| Eiding, H.; Kongsgaard, U. E.; Braarud, A.-C. Interhospital Transport of Critically Ill Patients: Experiences and Challenges, a Qualitative Study. Scand. J. Trauma Resusc. Emerg. Med. 2019, 27 (1), 27. | 1. Training and preparedness for intensive care transports  2.procedures  and checklists both before and during transport | Ⅰ-2.1 Qualification rate of the transport team assessment  Ⅰ-1.2 Established inter-hospital transport process and emergency plan |
| Borrows, E. L.; Lutman, D. H.; Montgomery, M. A.; Petros, A. J.; Ramnarayan, P. Effect of Patient- and Team-Related Factors on Stabilization Time during Pediatric Intensive Care Transport. Pediatr. Crit. Care Med. J. Soc. Crit. Care Med. World Fed. Pediatr. Intensive Crit. Care Soc. 2010, 11 (4), 451–456. | stabilization time | Ⅲ-3.2 Median transport stabilization time |
| Marsinyach Ros, I.; Sanchez García, L.; Sanchez Torres, A.; Mosqueda Peña, R.; Pérez Grande, M. D. C.; Rodríguez Castaño, M. J.; Elorza Fernández, M. D.; Sánchez Luna, M. Evaluation of Specific Quality Metrics to Assess the Performance of a Specialised Newborn Transport Programme. Eur. J. Pediatr. 2020, 179 (6), 919–928. | 1. Documented patient's family informed consent procedure  2. Favourable patient's clinical evolution  3. Mobilisation time  4. Patient's pain assessment and control  5. Accidental tracheal extubation, Occluded or dislodged tracheal tube | Ⅱ-2.1 Completeness rate of signing the inter-hospital transport informed consent  Ⅲ-4.1 Incidence of increase in disease assessment grade of patients after transport  Ⅲ-3.2 Median transport stabilization time  Ⅱ-3.4 Qualified rate of analgesic management  Ⅱ-3.5 Qualified rate of sedation management  Ⅲ-1.3 Incidence of technical problems |
| Jeyaraju, M.; Andhavarapu, S.; Palmer, J.; Bzhilyanskaya, V.; Friedman, E.; Lurie, T.; Patel, P.; Raffman, A.; Wang, J.; Tran, Q. K. Safety Matters: A Meta-Analysis of Interhospital Transport Adverse Events in Critically Ill Patients. Air Med. J. 2021, 40 (5), 350–358. | hypotension | * Incidence of hypotension during transport |
| Strauch, U.; Bergmans, D. C. J. J.; Habers, J.; Jansen, J.; Winkens, B.; Veldman, D. J.; Roekaerts, P. M. H. J.; Beckers, S. K. QUIT EMR Trial: A Prospective, Observational, Multicentre Study to Evaluate Quality and 24 Hours Post-Transport Morbidity of Interhospital Transportation of Critically Ill Patients: Study Protocol. BMJ Open 2017, 7 (3), e012861. | 1.24-hour post-transport mortality  2. Unstable situation with adequate intervention  3. requirements of transport team member  4. SOFA score pretransport | Ⅲ-4.2 Mortality within 24 hours of admission  Ⅱ-3.2 Correct handling rate of changes in condition during transport  Ⅰ-2.2 Qualification rate of transport team configuration  *Qualified rate of transport team qualifications  Ⅱ-1.1 Complete rate of initial assessment before driving |
| Gardiner, J.; McDonald, K.; Blacker, J.; Athikarisamy, S.; Sharp, M.; Davis, J. Unintended Events in Long-Distance Neonatal Interhospital Transport in Western Australia: A Comparison of Neonatal Specialist and Non-Neonatal Specialist Transport Teams. J. Pediatr. Clin. Pract. 2024, 11, 200102. | 1. Stabilization time  2. Team composition  3. Documented number of phone calls | Ⅲ-3.2 Median transport stabilization time  Ⅰ-2.2 Qualification rate of transport team configuration  Ⅱ-2.2 Efficient communication between transfer out - transport - receiving units |
| Staniszewska, A.; Gaba, K.; Patterson, B.; Wilson, S.; Bell, R.; Bicknell, C.; Brooks, M.; Callaway, M.; Goode, S.; Grier, S.; Hobson, A.; Mouton, R.; Neequaye, S.; Owens, G.; Rajakaruna, C.; Redfern, E.; Tsang, G.; Hinchliffe, R. Consensus Statement on the Interhospital Transfer of Patients with Acute Aortic Syndrome: TRAVERSING Delphi Study. Emerg. Med. J. EMJ 2024, 41 (3), 153–161. | 1. Vital sign monitoring during transfer | Ⅱ-3.1 Implementation rate of dynamic disease evaluation  *Rate of complete continuous vital sign records |
| Schwartz, H. P.; Bigham, M. T.; Schoettker, P. J.; Meyer, K.; Trautman, M. S.; Insoft, R. M. Quality Metrics in Neonatal and Pediatric Critical Care Transport: A National Delphi Project. Pediatr. Crit. Care Med. J. Soc. Crit. Care Med. World Fed. Pediatr. Intensive Crit. Care Soc. 2015, 16 (8), 711–717. | 1. Rate of cardiac arrest during transport  2. Recognition of abnormalities of vital signs and appropriate treatment  3. Use of a standardized patient care hand-off  4. Completeness of patient care documentation  5. Rate of transport  related patient  injuries | Ⅲ-1.1 Incidence of disease exacerbations  *Incidence of cardiac arrest during inter-hospital transport  Ⅱ-3.2 Correct handling rate of changes in condition during transport  Ⅱ-3.6 Completion rate of transport handover  Ⅱ-3.7 Qualified rate of medical documentation for transport  Ⅲ-1.2 Incidence of mobility problems |
| Chilakamarri P, Finn EB, Sather J, Sheth KN, Matouk C, Parwani V, et al. Failure Mode and Effect Analysis: Engineering Safer Neurocritical Care Transitions. Neurocrit Care. 2021,35(1):232-240. | No standard, universal notification of patient arrival | Ⅱ-2.2 Efficient communication between transfer out - transport - receiving units |
| Ground Air Medical Quality Transport Quality Improvement Collaborative. GAMUT Quality Improvement Consensus Metrics [EB/OL]. (2024-12-29) [2026-3-3]. | 1. Reliable pain assessments  2. Bedside time for transport patients  3. Percent of patient  contacts that undergo chart audit for  completion and accuracy.  4. Rate of transport-related patient injuries | Ⅱ-3.4 Qualified rate of analgesic management  Ⅱ-3.5 Qualified rate of sedation management  Ⅲ-3.2 Median transport stabilization time  Ⅱ-3.7 Qualified rate of medical documentation for transport  Ⅲ-1.2 Incidence of mobility problems |
| Expert Consensus Group on Interhospital Transport of Critically Ill Patients; National Center for Healthcare Quality Management in Emergency Medicine. Expert Consensus on Interhospital Transport of Critically Ill Patients. Chin. J. Emerg. Med. 2022, 31 (1), 17–23. | 1. Patient condition assessment and classification  2. Transport risk assessment and classification  3. Manpower staffing for transport  4. Preparation of transport equipment  5. Coordination and communication between referral institutions  6. Identification of the optimal transport route | Ⅱ-1.3 Accuracy rate of disease assessment classification  Ⅱ-1.4 Accuracy rate of transport risk assessment classification  Ⅰ-2.2 Qualification rate of transport team configuration  Ⅰ-1.1 Vehicle equipment and drug integrity rate  Ⅱ-2.2 Efficient communication between transfer out - transport - receiving units  Ⅱ-1.2 Planning rate of the best route for transport |
