# Supplementary material for: Developing sensitive quality indicators for ground inter-hospital transport of adult critically ill patients using Donabedian model
Source: Front Public Health. 2026 Apr 14;14:1744809. doi: 10.3389/fpubh.2026.1744809 (PMC13121257; doi:10.3389/fpubh.2026.1744809)
Supplement: Supplementary file 2 [file Table_2.doc]

# Appendix 2 Semi-structured qualitative interviews

A purposive sampling method was adopted to conduct semi-structured interviews with personnel from hospitals that have implemented inter-hospital transport (IHT) services by land and who possess experience in the quality management of IHT. Prior to the interviews, the researchers contacted the interviewees, explained the research purpose in detail, and scheduled interview times after obtaining their informed consent. The semi-structured interviews were conducted face-to-face or via remote audio and video by members of the research team who had received training in qualitative research courses, until data saturation was achieved. To ensure interview consistency, a unified set of interview questions was developed by the research team. Interviews were carried out in quiet offices or rest rooms.

# 1 Interview Guide

This interview aims to explore the key factors in quality management during IHT procedures. You may interrupt and withdraw from the study at any time during the interview. Your name will not be mentioned in future research reports. Please first provide your basic information, including age, gender, professional title, hospital grade, and duration of participation in inter-hospital transfer work.

# 2 Main Interview Questions

- Based on your experience in inter-hospital transport management, what do you believe are the core procedures/links of inter-hospital transfer?
- What was the most difficult challenge you encountered during inter-hospital transport?
- From which dimensions do you think the quality of inter-hospital transport should be evaluated? You may refer to dimensions such as structure, process, and outcome.

**3 Interviewee Characteristics**

**Table S2 General Characteristics of Interviewees**

| Code | Gender | Age (year) | Years of experience in IHT (year) | Position | Professional Title | Hospital Level |
| --- | --- | --- | --- | --- | --- | --- |
| 1 | Female | 38 | 11 | Head Nurse | Deputy Chief Nurse | Grade A Tertiary Hospital |
| 2 | Female | 32 | 7 | Nursing Team Leader | Supervising Nurse | Grade A Tertiary Hospital |
| 3 | Male | 52 | 15 | Department Director | Chief Physician | Grade A Tertiary Hospital |
| 4 | Female | 36 | 8 | Head Nurse | Supervising Nurse | Grade A Secondary Hospital |
| 5 | Male | 41 | 8 | Medical Team Leader | Attending Physician | Grade A Secondary Hospital |
| 6 | Male | 30 | 6 | Nursing Team Leader | Supervising Nurse | Grade A Tertiary Hospital |
| 7 | Female | 43 | 10 | Department Director | Chief Physician | Grade A Tertiary Hospital |
| 8 | Female | 34 | 7 | Nursing Team Leader | Supervising Nurse | Grade A Secondary Hospital |
