# Supplementary material for: Developing sensitive quality indicators for ground inter-hospital transport of adult critically ill patients using Donabedian model
Source: Front Public Health. 2026 Apr 14;14:1744809. doi: 10.3389/fpubh.2026.1744809 (PMC13121257; doi:10.3389/fpubh.2026.1744809)
Supplement: Supplementary file 3 [file Table_3.doc]

****Appendix 3 Definitions of third-level indicators****

**Table S3 Definitions and Calculation Formulas of Third-level indicators**

| **Third-level indicators** | **Definition or Connotation** | **Calculation formula** |
| --- | --- | --- |
| **Ⅰ-1.1 Develop inter-hospital transport procedures** | **IHT procedures help improve transport efficiency and ensure the standardized and orderly conduct of IHT.** | **A or B/1×100%, where A=1, indicating that there is an inter-hospital transport procedure; B=0: there is no inter-hospital transport procedure** |
| **Ⅰ-1.2 Formulate emergency plans for inter-hospital transport emergencies** | **The emergency plan covers scenarios such as changes in the patient's condition, equipment failure, transportation emergencies, and a sudden surge in the number of transport patients.** | **A or B/1×100%, where A=1, indicating that there is an emergency plan for inter-hospital transport emergencies; B=0: there is no emergency plan for inter-hospital transport emergencies** |
| **Ⅰ-2.1 Vehicle equipment integrity rate** | **Reflecting the quality of equipment management for IHT.**  **Note: Vehicle equipment shall be inspected for completeness, operational status, and secure fixation, etc.** | **Number of vehicle equipment integrity / Total number of vehicle transfer equipment × 100%** |
| **Ⅰ-2.2 Vehicle drug integrity rate** | **Reflecting the quality of drug management for IHT.**  **Note: Vehicle drugs shall be inspected for validity period, quantity, quality, proper storage, and integrity, etc.** | **Number of drugs with integrity in vehicle / Total number of drugs allocated for vehicle transport × 100%** |
| **Ⅰ-3.1 Qualified rate of examination of transport team members** | **Reflecting the mastery of professional theories and skills related to IHT among transport team members (including physicians, nurses, drivers, etc.), and serves as an important guarantee for the safety of IHT.**  **Note: The transport team shall be assessed at least once per quarter, including both theoretical and skill assessments. Passing the assessment is considered as having both the theoretical and practical skills scores qualified.** | **Number of transport team members qualified in examinations in the same period / Total number of participants in the assessment within the statistical period × 100%** |
| **Ⅰ-3.2 Qualified rate of hierarchical configuration of transport team members** | **Reflecting the effective human resource input of transport medical services and the extent to which the configuration matches the risk level of IHT.**  **Note: At least 1 physician and 1 nurse with corresponding qualifications and capabilities matching the patient's condition assessment grade shall be assigned, together with at least 1 driver. For transfers with a distance of 400 km or more, or a duration of 5 hours or more, two drivers shall be assigned.** | **Number of qualified vehicles of hierarchical configuration of transport team members in the same period / Total number of inter-hospital transport vehicles in the statistical period × 100%** |
| **Ⅱ-1.1 Accuracy rate of disease assessment classification** | **Disease assessment classification refers to the classification of patients' conditions based on vital signs, consciousness, respiratory support, circulatory support, clinical problems, and other related indicators.** | **Number of patients with correct disease assessment classification in the same period / Total number of transport patients in the statistical period × 100%** |
| **Ⅱ-1.2 Accuracy rate of transport risk assessment classification** | **Transport risk assessment classification is comprehensively determined based on the patient's disease assessment classification, transport mode, transport distance and duration, and serves as an important basis for the allocation of transport personnel, equipment, and drugs.** | **Number of patients with accurate transport risk assessment classification in the same period / Total number of transport patients in the statistical period × 100%** |
| **Ⅱ-2.1 Standard signing rate of informed consent for inter-hospital transport** | **Prior to IHT, patients or their family members shall be fully informed of the disease condition, transport risks, estimated transport duration, destination, professional qualifications of the transport team, bed arrangement at the receiving hospital, and estimated costs. The benefits and risks of transport shall be fully analyzed and explained. The Informed Consent Form for IHT serves as a key document verifying whether communication and risk disclosure have been conducted.**  **Note: The signing of informed consent is deemed non-standard in the following situations: incomplete completion of the informed consent form, such as missing signature date, risk notification content, or physician's signature, etc.; insufficient notification of transport risks, transport costs, and other relevant information; signing by the patient in an unconscious or sedated state; failure to indicate the relationship between the proxy signer and the patient, etc.** | **Number of patients with standard signed informed consent for inter-hospital transport in the same period / Total number of transport patients in the statistical period × 100%** |
| **Ⅱ-3.1 Implementation rate of dynamic disease evaluation** | **Dynamic disease evaluation refers to the continuous monitoring and assessment of heart rate/pulse, blood pressure, respiration, oxygen saturation, etc., as well as the documentation of changes in the patient's condition.** | **Number of patients undergoing dynamic disease evaluation in the same period / Total number of transport patients in the statistical period × 100%** |
| **Ⅱ-3.2 Effective treatment rate of changes in condition during transport** | **Reflecting whether the transport team has effectively managed changes in the patient's condition during transport.** | **Number of cases with effective management of condition changes during transport in the statistical period/ Total number of cases with condition changes during transport in the statistical period × 100%** |
| **Ⅱ-3.3 Accurate execution rate of medical orders** | **Accurate execution of medical orders includes the accurate implementation of medication dosage and administration route, dosage adjustment, oxygen therapy adjustment, and other related nursing procedures.** | **Number of accurately executed medical orders in the same period / Total number of medical orders executed in the statistical period × 1000‰** |
| **Ⅱ-3.4 The implementation rate of standardized handover** | **Standardized handover shall be implemented at each link of transport using a standardized handover form to ensure accurate information transmission. The standardized handover form should include primary condition, monitoring data during transport, special events and treatments, pipelines, medications, and information of the transporting physician, etc.**  **Note: The implementation of standardized handover is defined as having complete and properly filled standardized handover records.** | **Number of patients with standardized handover to the transferring and receiving hospitals in the same period / Total number of transport patients in the statistical period × 100%** |
| **Ⅱ-3.5 Qualified rate of medical documentation for transport** | **Medical documentation for transport includes physicians' medical records and nurses' nursing records.** | **Number of cases with qualified medical documentation for transport in the same period / Total number of transported cases in the statistical period × 100%** |
| **Ⅲ-1.1 Incidence of accidental catheter displacement** | **Catheters include artificial airway, central venous catheter, thoracic or abdominal drainage tube, indwelling gastric tube, etc. Accidental catheter displacement includes both accidental displacement and accidental extubation. One transport case may involve one or more episodes of catheter displacement.** | **Number of accidental catheter displacement or dislodgement events during transport in the same period / Total number of transport patients carrying catheters in the statistical period × 1000‰** |
| **Ⅲ-1.2 Incidence of medical equipment failure during transport** | **Medical equipment failure refers to insufficient battery or other malfunctions of instruments such as ventilators, insufficient oxygen supply, disconnection of oxygen connectors, disconnection between endotracheal tube and ventilator, etc. One transport case may involve one or more medical equipment failure event.** | **frequency of medical equipment failures in transport in the same period / Total number of transport patients in the statistical period × 1000‰** |
| **Ⅲ-1.3 Incidence of patient accidental injuries during transport** | **Patient accidental injuries during transport include traffic accidents, injuries caused by improper driving, falls from the bed during transport, and other complications, etc.** | **Number of patients with accidental injury during transport in the same period / Total number of transport patients in the statistical period × 1000‰** |
| **Ⅲ-2.1 Patient satisfaction with transport medical services** | **It reflects the extent to which IHT medical services meet patients' expectations.**  **Note: Participants rated the inter-hospital transport medical service on a scale from 1 to 10, where 1 indicated extremely dissatisfied and 10 indicated extremely satisfied.** | **Survey scores of inter-hospital transport patients' satisfaction with transport medical services** |
| **Ⅲ-3.1 Average time taken to prepare for transport** | **Transport preparation time refers to the interval from receiving the transport request call to vehicle departure. It is an important indicator reflecting the response speed and preparation efficiency of IHT.**  **Note: Transport preparation time is measured in minutes.** | **Total transport preparation time in the same period / Total number of transport patients in the statistical period** |
| **Ⅲ-3.2 Median transport stabilization time** | **Transport stabilization time refers to the interval from the arrival of the transport team at the patient's bedside to departure from the referring hospital, including bedside handover, patient assessment and intervention, patient transport, equipment replacement and stabilization, as well as communication with the patient or family members.**  **Note: Transport stabilization time is measured in minutes.** | **X _(n+1)_ /2, n is odd**  **(X_n/2_+X_n/2+1_)/2, n is even**  **Note: n为转运患者数，X为转运稳定时间**  **Note: n represents the number of transported patients, and X represents the transport stabilization time.** |
| **Ⅲ-3.3 Rate of compliance with the expected arrival time of transport** | **Expected arrival time refers to the estimated time of arrival at the receiving hospital. Note: Actual transport time exceeding 15% of the estimated transport time is regarded as non-compliant. The transport time is measured in minutes.** | **Number of vehicles with the expected arrival time of the receiving hospital in the same period / Total number of inter-hospital transport vehicles in the statistical period ×100%** |
| **Ⅲ-4.1 Rate of transport patients smoothly arrive** | **This indicator is used to evaluate the rate of changes in the patient's condition before and after transport. It reflects the transport team's ability in benefit-risk assessment before transport and the quality of medical care during IHT.**  **Note: "Smoothly arrive" means that the patient's condition after transport is unchanged or improved compared with that before transport.** | **Number of patients whose condition was stable or improved after transport in the same period / Total number of transport patients in the statistical period ×100%** |
